# Association between the platelet-to-albumin ratio and 28-day all-cause mortality in critically ill patients with Pulmonary embolism: a retrospective cohort study and predictive model establishment based on machine learning

**DOI:** 10.3389/fmed.2025.1680205

**Published:** 2025-10-24

**Authors:** Danyang Chang, Fuhong Zheng, Lei Zhu, Haibo Liu

**Affiliations:** Department of Emergency, The First Hospital of Jilin University, Changchun, Jilin, China

**Keywords:** pulmonary embolism, platelet-to-albumin ratio, critical illness, Boruta algorithm, machine learning

## Abstract

**Background:**

Pulmonary embolism (PE) is a serious condition that is frequently encountered in clinical practice. It has been demonstrated that the body’s physiological responses to platelet activation can lead to significant complications, including pulmonary hypertension, bronchoconstriction, and right heart failure. Albumin is recognized as a composite indicator of acute-phase reactant proteins, which have osmotic and anti-inflammatory properties, as well as nutrient and metabolic imbalance. Albumin demonstrates independent prognostic value in a variety of diseases. The platelet-to-albumin ratio (PAR) has emerged as a reliable predictor of mortality and complications based on systemic inflammation in a number of diseases. However, studies on the relationship between PAR and adverse outcomes in critically ill patients with pulmonary embolism are limited. Thus, this study aimed to investigate whether PAR could be a useful indicator for assessing pulmonary embolism outcomes.

**Methods:**

The clinical data of 1163 patients with critical pulmonary embolisms were extracted from the MIMIC-IV (version 2.2) database. The study population was categorized into four groups according to PAR quartiles. The primary regression was 28-day ICU mortality, while the secondary regressions were 7-d and 14-d ICU mortality. Restricted cubic splines, Cox proportional hazards regression, and Kaplan-Meier curves were used to explore the relationship between PAR and adverse outcomes. We assessed the predictive power of PAR using the Boruta algorithm and built predictive models using machine learning algorithms.

**Results:**

Data from 1163 patients diagnosed with pulmonary embolism were analyzed. Lower PAR was significantly associated with an increased risk of 7-d (*p* < 0.001), 14-d (*p* < 0.001), and 28-d (*p* < 0.005) ICU mortality compared with higher PAR. The restricted cubic spline curve revealed an “L-shaped” relationship between PAR and survival, suggesting that an increase in PAR is linked to a reduced risk of adverse events. Patients with lower PAR had a higher risk of death within 7, 14, and 28 days in the ICU compared to those with higher PAR (*p* < 0.05). Boruta feature selection showed PAR had a higher Z score, and the model built using the Conditional Inference Trees algorithm had the best performance (AUC = 0.623).

**Conclusion:**

PAR showed an “L”-shaped relationship with all-cause mortality at 7, 14, and 28 days in critically ill patients. Low PAR was significantly associated with an increased risk of adverse events, suggesting that PAR may be a predictor of adverse outcomes in patients with pulmonary embolisms.

## Introduction

As the most critical clinical manifestation of venous thromboembolism (VTE), pulmonary embolism (1) (PE) continues to pose a significant global public health challenge due to its high morbidity, insidious onset, and potential lethality.(2) According to the latest epidemiological studies, PE is the third leading cause of cardiovascular death, with an annual incidence rate of 0.5–1.0 per 1,000 people. More than 50% of cases go undiagnosed prior to diagnosis, highlighting the serious challenge of early diagnosis. In the acute phase of PE, platelets (3) not only contribute to the growth of blood clots, but also promote endothelial injury and microcirculatory impairment in the pulmonary vasculature by releasing inflammatory mediators (PF4 (4) and sCD40L, which lead to endothelial damage and microcirculatory disorders. Albumin, the major serum protein, is also recognized as an acute-phase reactant protein (5) with osmotic and anti-inflammatory properties. Low albumin levels are a known correlate of severity in many pathologies, including atrial fibrillation (6, 7). Recently, the platelet-to-albumin ratio (8) (PAR) has been identified as a potential prognostic biomarker for various diseases, including IgA nephropathy (9, 10), cholangiocarcinoma (11), nasopharyngeal carcinoma (12), peritoneal dialysis (13), and new-onset atrial fibrillation in patients with ST-segment elevation myocardial infarction (14). However, the relationship between PAR and prognosis in patients with pulmonary embolism remains unclear.

This study retrospectively enrolled patients diagnosed with pulmonary embolism between January 2020 and October 2022. Baseline demographic and clinical data were collected for each patient, including platelet count and albumin levels, and the platelet-to-albumin ratio (PAR) was calculated. Kaplan-Meier survival analysis and multivariate Cox regression analysis were employed to predict 7-d,14-d and 28-d mortality in pulmonary embolism patients.

## Materials and methods

The MIMIC-IV (v2.2) database is an open-access repository created by the MIT Laboratory for Computational Physiology, accessible via its official website: https://mimic.mit.edu/. This database serves as a valuable resource for clinical decision support, predictive modeling, and critical care research, containing de-identified data from over 50,000 ICU patients admitted to Beth Israel Deaconess Medical Center between 2008 and 2019. The data utilized in this study were obtained from this source, with Institutional Review Board approval obtained for database access, and a waiver of informed consent was granted for its use. The author (YDC) obtained access to the database (certificate number: 65992548).

Patient data with acute pancreatitis (APE) were extracted from the MIMIC-IV database using International Classification of Diseases, Ninth Revision (ICD-9) code 415 and International Classification of Diseases, Tenth Revision (ICD-10) code 126. This study included patients aged between 18 and 90 years with first-time ICU admission who survived ≥3 days after hospitalization. Exclusion criteria comprised:

1. Patients with hepatitis, cirrhosis, malignancy, type 1 diabetes with diabetic nephropathy, primary thrombocythemia, or chronic kidney disease (CKD)
2. ICU length of stay <24 h
3. Missing platelet (Plt) and albumin (Alb) measurements within 24 h of admission

## Outcome

The primary outcome was 28-day all-cause mortality, and the secondary outcome was 7-d,14-d all-cause mortality.

## Data extraction

Data extraction was performed using DecisionLinnc (15). software. Patient characteristics including age, sex, body weight, and ethnicity were collected. Comorbidity information was extracted based on International Classification of Diseases (ICD) codes, encompassing hypertension, type 2 diabetes mellitus (T2DM), type 1 diabetes mellitus (T1DM), pulmonary tuberculosis, pneumonia, stroke, hyperlipidemia, chronic bronchitis, heart failure, myocardial infarction, coronary artery disease (CAD), chronic obstructive pulmonary disease (COPD), and coronavirus infection.

Vital signs comprised heart rate, partial pressure of carbon dioxide (PCO_2_), partial pressure of oxygen (PO_2_), arterial oxygen pressure (PaO_2_), and arterial oxygen saturation (SaO_2_). Laboratory parameters included hematocrit (Hct), white blood cell count (WBC), platelet count (PLT), albumin, hemoglobin, red blood cell distribution width (RDW), lactate, prothrombin time (PT), Alanine Aminotransferase (ALT), Aspartate Aminotransferase (AST), blood urea nitrogen (BUN), serum creatinine, serum glucose, and N-terminal pro-B-type natriuretic peptide (NT-proBNP).

Disease severity was assessed using the Sequential Organ Failure Assessment (SOFA) score and Pulmonary Embolism Severity Index (PESI). The platelet-to-albumin ratio (PAR) (8) was calculated for prognostic evaluation.

### Statistical analysis

As the current study was a retrospective analysis, no sample size calculation was performed. Variables with missing data rates exceeding 20% were excluded, while multiple imputation was applied to variables with missing data rates below 20%. Patients were stratified into four groups based on PAR quartiles. Normally distributed continuous variables were expressed as mean (standard deviation [SD]) and analyzed using analysis of variance (ANOVA). Non-normally distributed variables were analyzed using the Mann-Whitney U test or Kruskal-Wallis test. Categorical variables were presented as numbers and percentages, and analyzed using the χ^2^ test or Fisher’s exact test. Kaplan-Meier survival curves with log-rank tests were used to compare 7-d,14-d,28-d survival rates across the four groups. Hazard ratios (HRs) and 95% confidence intervals (95% CIs) were evaluated using proportional hazards regression models (Cox regression models). Model I did not adjust for covariates. Model II adjusted for age, sex, and ethnicity. Model III further adjusted for additional relevant variables. Univariate Cox regression analysis was used to screen potential risk factors, and variables with *p*-values < 0.1 were included in subsequent multivariate Cox regression analysis. Restricted cubic spline (RCS) analysis was employed to assess non-linear associations of PAR. Subgroup analyses were conducted to examine the consistency of PAR effects across subgroups, with results presented as forest plots.

### Restricted cubic splines

In this study, we collected data on survival (the outcome variable); the PAR (continuous predictor variable); and age, weight, sex and race. Potential linear relationships between the change in the PAR and survival were examined by a Cox regression model with restricted cubic spline (RCS). The model with the lowest Akaike information criterion value was selected for the RCS.

### Subgroup analysis

A subgroup analysis was conducted based on prespecified criteria, including age, sex, and race, and univariate analysis were performed. The univariate analysis was adjusted for WBC, Hematocrit, Hemoglobin, RDW, Glucose, SOFA scores and PESI scores. Patients were stratified into two groups based on age (< 65 years and ≥ 65 years). Cox proportional hazards regression analysis was performed for each subgroup, and the results were visually presented using forest plots, illustrating hazard ratios (HRs) and 95% confidence intervals (CIs).

### Establishment and validation of the prediction models

Boruta’s algorithm is a method for identifying the most important features in a dataset. It identifies importance by comparing the *Z*-value (16) of each feature with that of its corresponding “shadow feature.” In the algorithm, all real features are copied and shuffled. Then, the Z-value of each feature is obtained from the Random Forest model. If a real feature’s Z-value is significantly higher than the maximum Z-value of its shadow feature in multiple independent tests, the real feature is labeled “important” (red area), also known as an acceptable variable. Otherwise, it is labeled as “unimportant” (green area), also known as an unacceptable variable. Acceptable variables are retained during the feature selection process because they contribute to the model’s performance. Unacceptable variables are excluded from the final selection because they do not demonstrate predictive power for the target variable. Additionally, acceptable variables are incorporated into the machine learning algorithm. The following were used to predict the risk of death at 28 days in patients with pulmonary embolism: Conditional Inference Tree, Conditional Inference Tree survival learner, GBM (Gradient Boosted Tree) survival learner, Random Forest Time, Random Forest survival learner, SVM (Support Vector Machine) survival learner, and XGBoost (Extreme Gradient Boosted) survival learner. Hyperparameter tuning was performed during the machine learning model-building process for model building and evaluation. The ROC curve and its corresponding area under the curve (AUC) were used to evaluate model performance.

## Results

### Baseline characteristics

**FIGURE 1:**
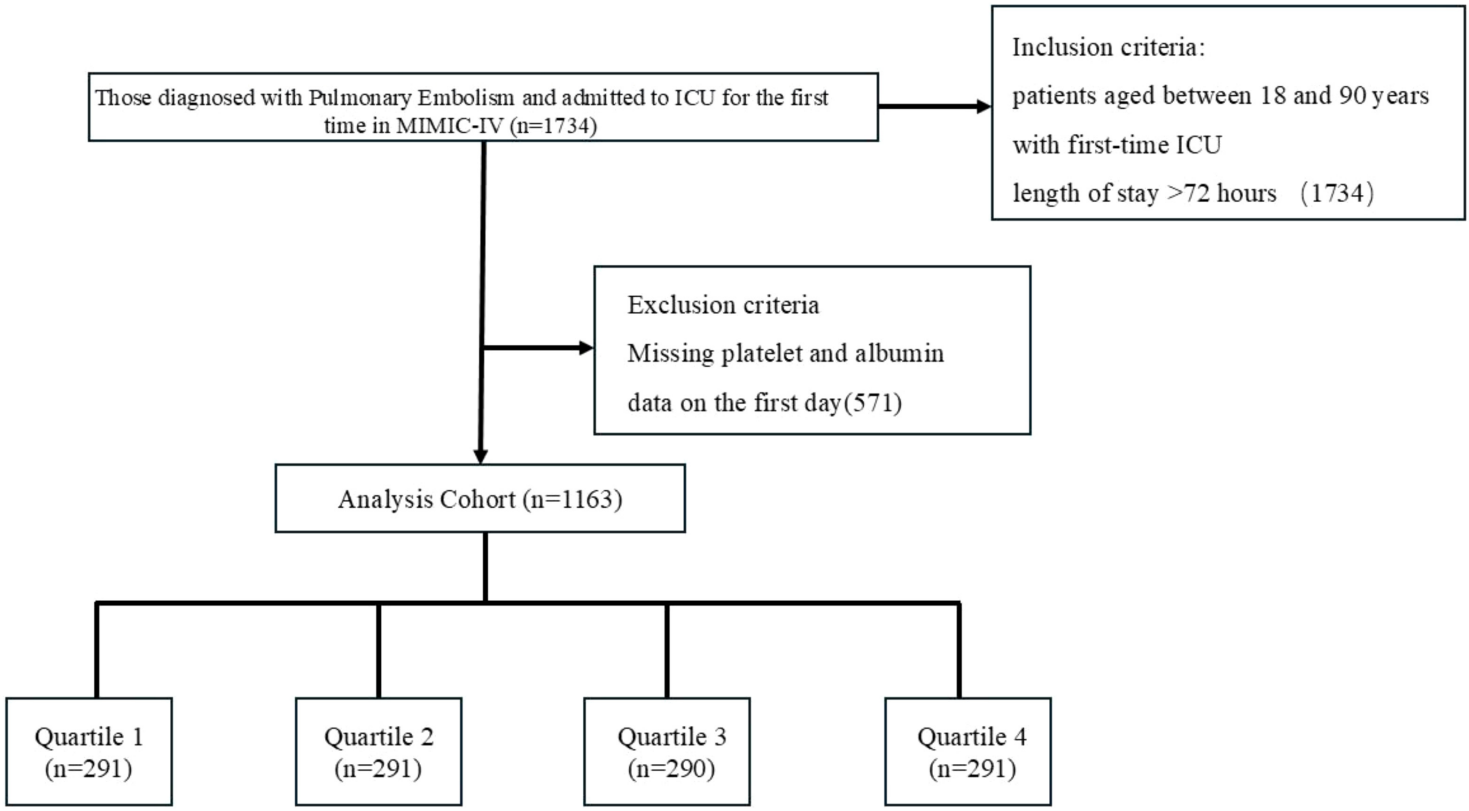
Selection of the study population from the MIMIC-IV database.

Data on 1163 patients diagnosed with pulmonary embolism were extracted from MIMIC-IV (Figure 1). Table 1 shows the baseline characteristics of the study population. The population included 605 males (52.02%), 438 patients with hypertension (37.66%), 28 patients with tuberculosis (2.41%), 489 patients with pneumonia (42.05%), 87 patients with stroke (7.48%), 285 patients with type 2 diabetes mellitus (24.51%), 17 patients with type 1 diabetes mellitus (1.46%), and 305 patients with hyperlipidemia (26.%), 106 patients with chronic bronchitis (9.11%), 302 patients with heart failure (25.97%), 92 patients with myocardial infarction (7.91%), 282 patients with coronary artery disease (24.25%), 192 patients with chronic obstructive pulmonary disease (16.51%), and 29 patients with coronary artery disease (2.49%). Patients were categorized into four groups according to quartile boxes: 291 patients in quartiles 1, 2, and 4; 290 patients in quartile 3. Patients in quartile 4 had higher WBC, Lactate, Glucose, pCO_2_, pO_2_, SOFA scores and heart rates, as well as lower ages, hematocrit levels, hemoglobin levels, Creatinine, PT, erythrocyte distribution width and PESI scores.

**TABLE 1:** Patient demographics and baseline characteristics.

	PAR					
Characteristic (mean ± SD)	Overall	Q1	Q2	Q3	Q4	*p*-value
	*N* = 1,163	*N* = 291	*N* = 291	*N* = 290	*N* = 291	
**Laboratory indicators**						
Weight	85.35 ± 28.99	86.18 ± 28.38	85.70 ± 29.16	86.15 ± 27.16	83.36 ± 31.17	0.165
PAR	163.57 ± 118.45	61.75 ± 18.06	111.88 ± 13.00	166.17 ± 20.18	314.49 ± 139.20	<0.001
WBC	12.21 ± 7.79	10.27 ± 7.18	11.33 ± 6.66	12.75 ± 8.21	14.50 ± 8.35	<0.001
Hematocrit	32.66 ± 7.15	33.45 ± 7.38	33.13 ± 6.92	32.62 ± 7.17	31.45 ± 6.98	<0.05
Hemoglobin	10.58 ± 2.38	10.86 ± 2.38	10.71 ± 2.33	10.57 ± 2.40	10.18 ± 2.38	<0.05
RDW	15.67 ± 2.63	15.82 ± 2.54	15.82 ± 2.92	15.71 ± 2.80	15.32 ± 2.18	0.219
Glucose	151.57 ± 90.72	148.35 ± 72.43	153.76 ± 101.45	149.69 ± 69.98	154.49 ± 111.94	0.932
Lactate	2.20 ± 1.83	2.28 ± 1.76	2.10 ± 1.72	2.11 ± 1.54	2.32 ± 2.22	0.249
Pco_2_	43.91 ± 13.89	43.95 ± 15.49	43.99 ± 12.37	43.57 ± 14.95	44.13 ± 12.52	0.728
Po_2_	108.24 ± 90.95	102.33 ± 91.44	107.86 ± 96.07	105.50 ± 84.74	117.24 ± 90.95	<0.05
PT	16.33 ± 7.67	16.07 ± 5.93	17.06 ± 10.08	16.49 ± 8.33	15.71 ± 5.38	0.559
ALT	100.73 ± 320.63	108.10 ± 307.66	104.50 ± 351.78	107.80 ± 376.09	82.54 ± 228.28	0.344
AST	145.71 ± 524.73	156.73 ± 472.31	150.36 ± 558.97	143.25 ± 602.98	132.49 ± 452.85	0.621
Creatinine	1.34 ± 1.64	1.65 ± 2.63	1.12 ± 0.74	1.30 ± 1.24	1.31 ± 1.30	<0.05
Urea nitrogen	25.81 ± 21.21	27.83 ± 23.13	24.02 ± 17.73	25.90 ± 22.31	25.49 ± 21.21	0.131
SOFA	4.99 ± 3.67	5.24 ± 3.97	4.39 ± 3.24	4.94 ± 3.50	5.37 ± 3.87	<0.05
PESI	120.89 ± 27.10	120.31 ± 27.11	120.42 ± 29.06	123.17 ± 25.92	119.68 ± 26.20	0.492
Heart rate	98.41 ± 22.45	94.68 ± 20.91	96.97 ± 22.03	99.11 ± 23.69	102.89 ± 22.40	<0.001
NBPS	121.75 ± 23.29	122.36 ± 21.40	121.95 ± 23.66	121.11 ± 23.59	121.58 ± 24.49	0.953
**Demographics**						
Age	61.86 ± 16.21	65.14 ± 14.77	63.58 ± 16.27	61.75 ± 15.53	56.99 ± 17.08	<0.001
Gender						0.785
Female	558.00 (47.98%)	132.00 (45.36%)	142.00 (48.80%)	142.00 (48.97%)	142.00 (48.80%)	
Male	605.00 (52.02%)	159.00 (54.64%)	149.00 (51.20%)	148.00 (51.03%)	149.00 (51.20%)	
RACE						0.580
Black	150.00 (12.90%)	34.00 (11.68%)	37.00 (12.71%)	42.00 (14.48%)	37.00 (12.71%)	
Other	298.00 (25.62%)	64.00 (21.99%)	81.00 (27.84%)	74.00 (25.52%)	79.00 (27.15%)	
White	715.00 (61.48%)	193.00 (66.32%)	173.00 (59.45%)	174.00 (60.00%)	175.00 (60.14%)	
**Comorbidities**						
Hypertension						0.564
No	725.00 (62.34%)	179.00 (61.51%)	175.00 (60.14%)	180.00 (62.07%)	191.00 (65.64%)	
Yes	438.00 (37.66%)	112.00 (38.49%)	116.00 (39.86%)	110.00 (37.93%)	100.00 (34.36%)	
Pulmonary tuberculosis						0.360
No	1,135.00 (97.59%)	286.00 (98.28%)	285.00 (97.94%)	284.00 (97.93%)	280.00 (96.22%)	
Yes	28.00 (2.41%)	5.00 (1.72%)	6.00 (2.06%)	6.00 (2.07%)	11.00 (3.78%)	
Pneumonia						<0.001
No	674.00 (57.95%)	201.00 (69.07%)	182.00 (62.54%)	153.00 (52.76%)	138.00 (47.42%)	
Yes	489.00 (42.05%)	90.00 (30.93%)	109.00 (37.46%)	137.00 (47.24%)	153.00 (52.58%)	
Stroke						0.936
No	1,076.00 (92.52%)	269.00 (92.44%)	267.00 (91.75%)	270.00 (93.10%)	270.00 (92.78%)	
Yes	87.00 (7.48%)	22.00 (7.56%)	24.00 (8.25%)	20.00 (6.90%)	21.00 (7.22%)	
Diabetes II						0.061
No	878.00 (75.49%)	203.00 (69.76%)	222.00 (76.29%)	224.00 (77.24%)	229.00 (78.69%)	
Yes	285.00 (24.51%)	88.00 (30.24%)	69.00 (23.71%)	66.00 (22.76%)	62.00 (21.31%)	
Diabetes I						0.765
No	1,146.00 (98.54%)	288.00 (98.97%)	287.00 (98.63%)	284.00 (97.93%)	287.00 (98.63%)	
Yes	17.00 (1.46%)	3.00 (1.03%)	4.00 (1.37%)	6.00 (2.07%)	4.00 (1.37%)	
Hyperlipidemia						0.217
No	858.00 (73.77%)	206.00 (70.79%)	210.00 (72.16%)	215.00 (74.14%)	227.00 (78.01%)	
Yes	305.00 (26.23%)	85.00 (29.21%)	81.00 (27.84%)	75.00 (25.86%)	64.00 (21.99%)	
Chronic bronchitis						0.069
No	1,057.00 (90.89%)	256.00 (87.97%)	262.00 (90.03%)	265.00 (91.38%)	274.00 (94.16%)	
Yes	106.00 (9.11%)	35.00 (12.03%)	29.00 (9.97%)	25.00 (8.62%)	17.00 (5.84%)	
Heart failure						<0.05
No	861.00 (74.03%)	210.00 (72.16%)	205.00 (70.45%)	212.00 (73.10%)	234.00 (80.41%)	
Yes	302.00 (25.97%)	81.00 (27.84%)	86.00 (29.55%)	78.00 (26.90%)	57.00 (19.59%)	
Myocardial infarction						0.578
No	1,071.00 (92.09%)	268.00 (92.10%)	272.00 (93.47%)	268.00 (92.41%)	263.00 (90.38%)	
Yes	92.00 (7.91%)	23.00 (7.90%)	19.00 (6.53%)	22.00 (7.59%)	28.00 (9.62%)	
Coronary artery disease						0.882
No	881.00 (75.75%)	217.00 (74.57%)	218.00 (74.91%)	223.00 (76.90%)	223.00 (76.63%)	
Yes	282.00 (24.25%)	74.00 (25.43%)	73.00 (25.09%)	67.00 (23.10%)	68.00 (23.37%)	
COPD						<0.001
No	971.00 (83.49%)	233.00 (80.07%)	235.00 (80.76%)	238.00 (82.07%)	265.00 (91.07%)	
Yes	192.00 (16.51%)	58.00 (19.93%)	56.00 (19.24%)	52.00 (17.93%)	26.00 (8.93%)	
Coronavirus						0.252
No	1,134.00 (97.51%)	288.00 (98.97%)	282.00 (96.91%)	280.00 (96.55%)	284.00 (97.59%)	
Yes	29.00 (2.49%)	3.00 (1.03%)	9.00 (3.09%)	10.00 (3.45%)	7.00 (2.41%)	

WBC, white blood cell count; RDW, red blood cell distribution width; PLT, platelet count; PaCO_2_, carbon dioxide pressure; PaO_2_**,** arterial oxygen pressure; AST, aspartate aminotransferase; SOFA, sequential organ failure assessment; Diabetes I, type 1 diabetes; Diabetes II, type 2 diabetes; PAR, platelet-to-albumin ratio.

## Clinical outcomes

**FIGURE 2:**
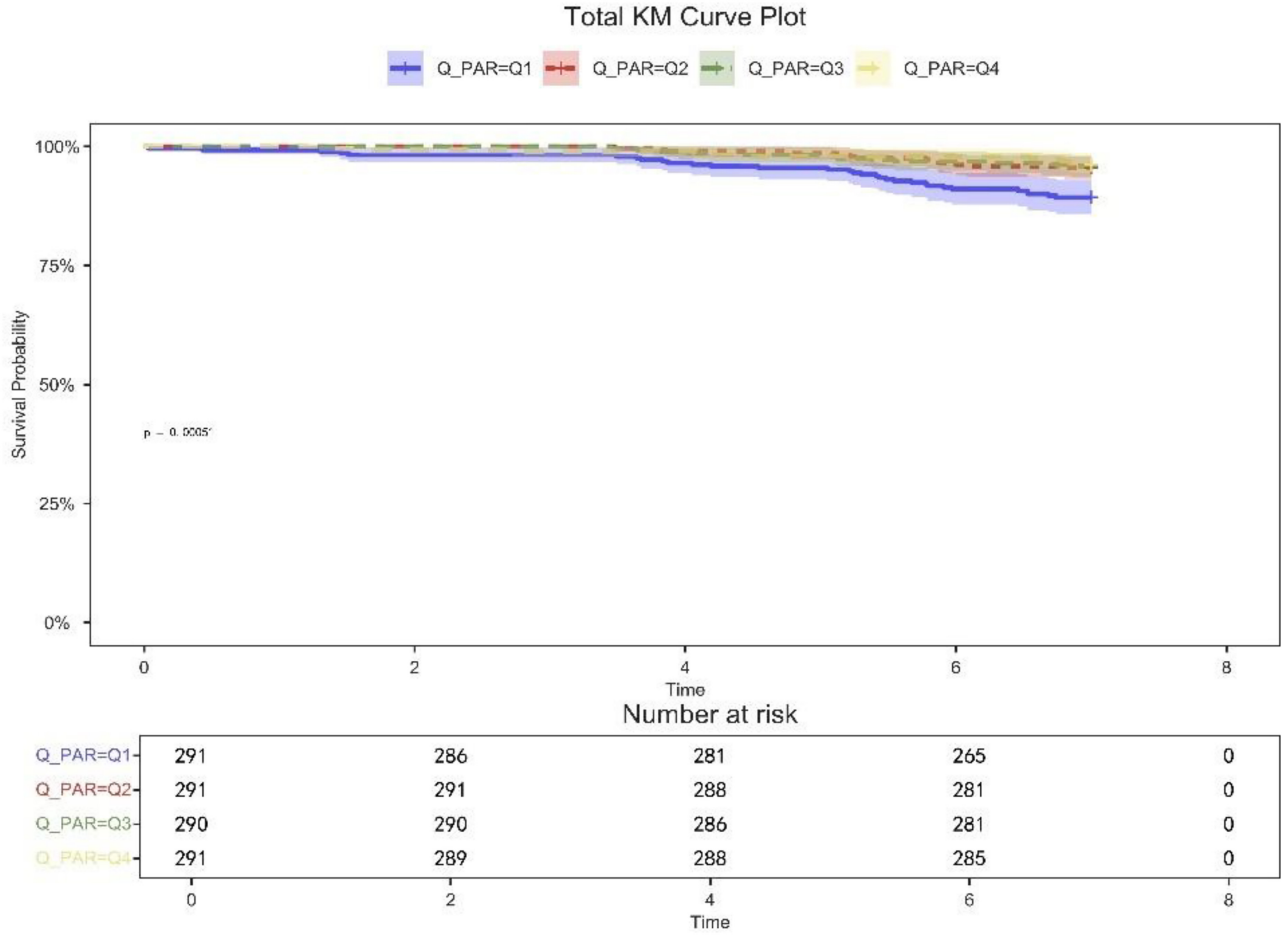
7-day KM survival curve. KM curves showing the survival rates at 7 days for each quartile.

**FIGURE 3:**
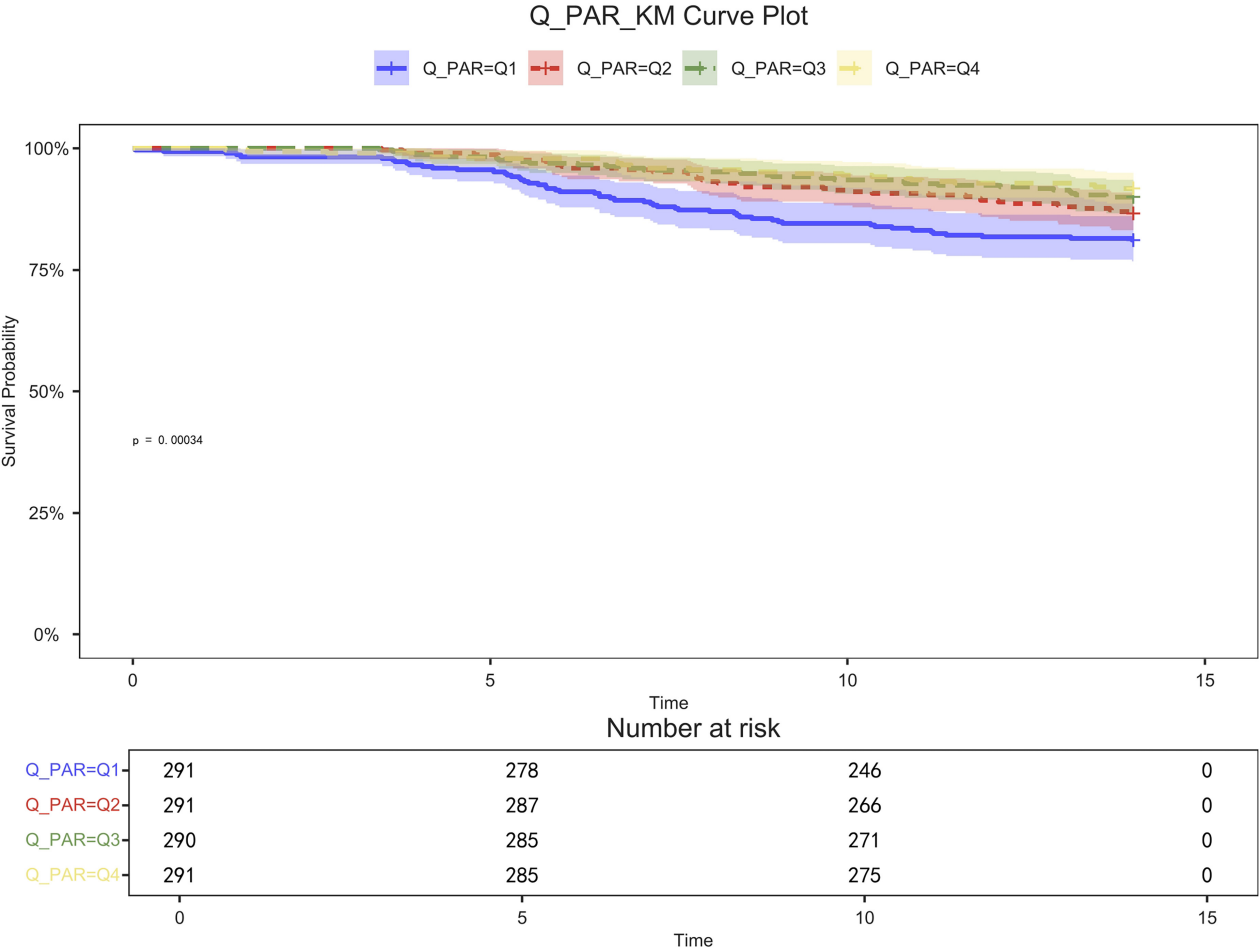
14-day KM survival curve. KM curves showing the survival rates at 14 days for each quartile.

**FIGURE 4:**
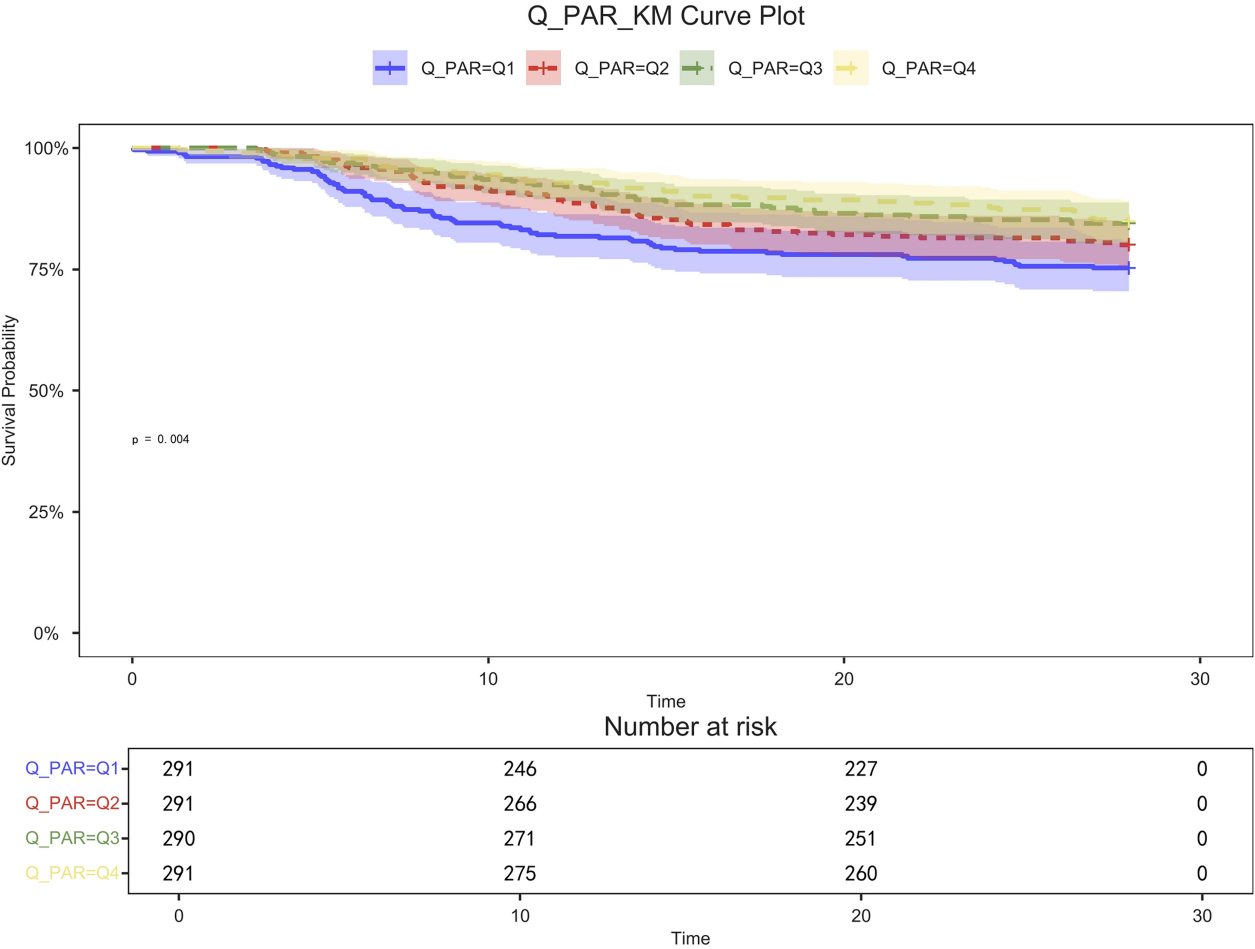
28-day KM survival curve. KM curves showing the survival rates at 28 days for each quartile.

Table 2 shows that quartile 3 and 4 had the highest survival rates for 7-d, 14-d, and 28-d ICU stays. The Kaplan-Meier curve revealed that the mortality rate was notably higher in the low PAR group compared to the other groups (*p* < 0.001) (Figures 2–4).

**TABLE 2:** 7-day,14-day,28-day all-cause mortality.

Variable	Levels	N	Overall	Q1	Q2	Q3	Q4	*p*-value
			*N* = 1,163	*N* = 291	*N* = 291	*N* = 290	*N* = 291	
7-day mortality, *n* (%)	No	1163						<0.001
			1,016.00 (87.36%)	236.00 (81.10%)	252.00 (86.60%)	261.00 (90.00%)	267.00 (91.75%)	
	Yes		147.00 (12.64%)	55.00 (18.90%)	39.00 (13.40%)	29.00 (10.00%)	24.00 (8.25%)	
14-day mortality, *n* (%)	No	1163						<0.001
			1,096.00 (94.24%)	260.00 (89.35%)	278.00 (95.53%)	278.00 (95.86%)	280.00 (96.22%)	
	Yes		67.00 (5.76%)	31.00 (10.65%)	13.00 (4.47%)	12.00 (4.14%)	11.00 (3.78%)	
28-day mortality, *n* (%)	No	1163						0.009
			944.00 (81.17%)	219.00 (75.26%)	233.00 (80.07%)	245.00 (84.48%)	247.00 (84.88%)	
	Yes		219.00 (18.83%)	72.00 (24.74%)	58.00 (19.93%)	45.00 (15.52%)	44.00 (15.12%)	

Patients in quartile 1 had the lowest survival rates at 7,14,28 days, and the difference was statistically significant (Figures 2–4).

## Restricted cubic spline

**FIGURE 5:**
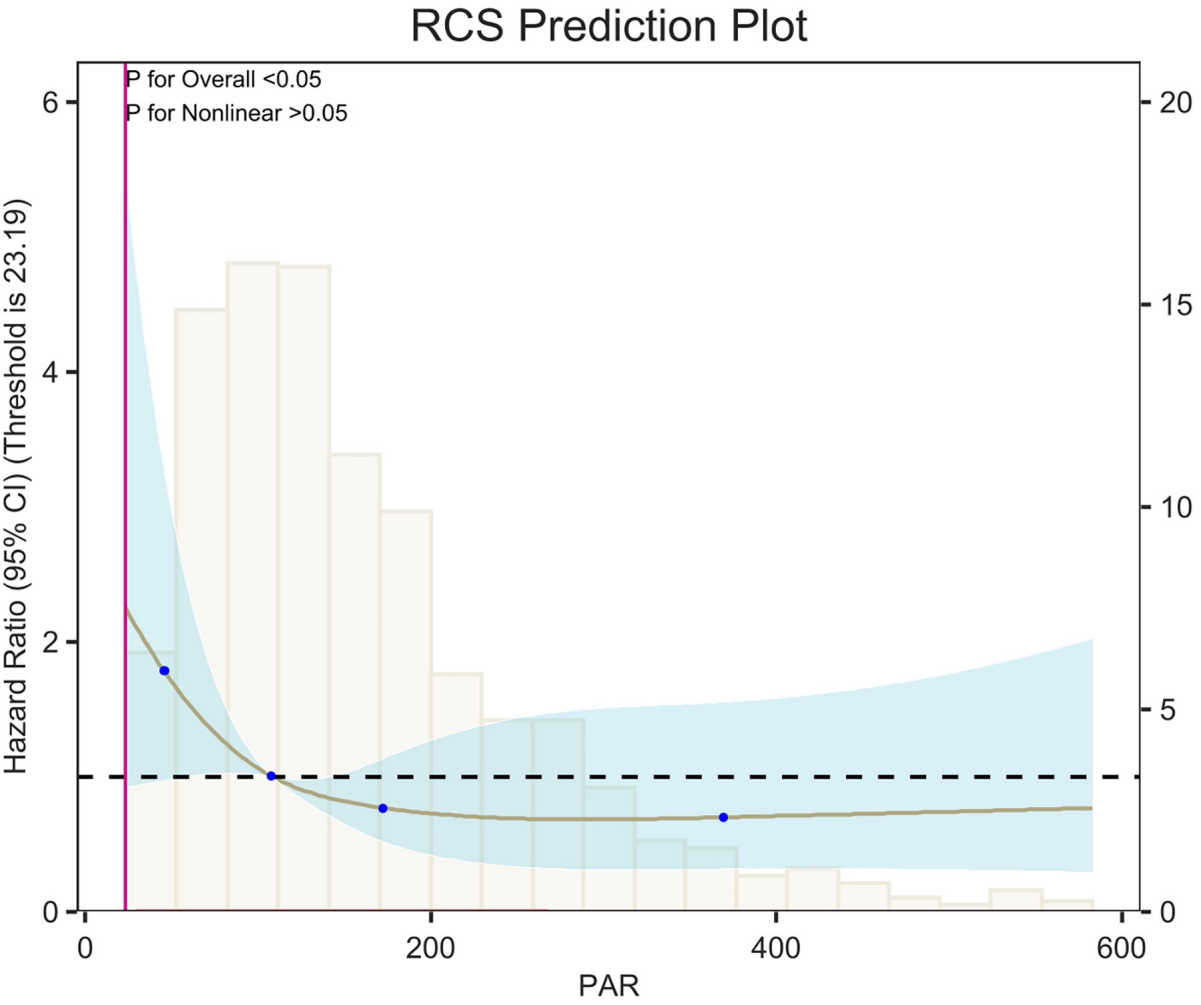
Restricted cubic spline (RCS) analysis of 7-day all-cause mortality. The curves show the adjusted estimated risk ratios, and the shaded bands show the 95% confidence intervals. The horizontal dashed line indicates a risk ratio of 1.0. HR, hazard ratio; CI, confidence interval.

**FIGURE 6:**
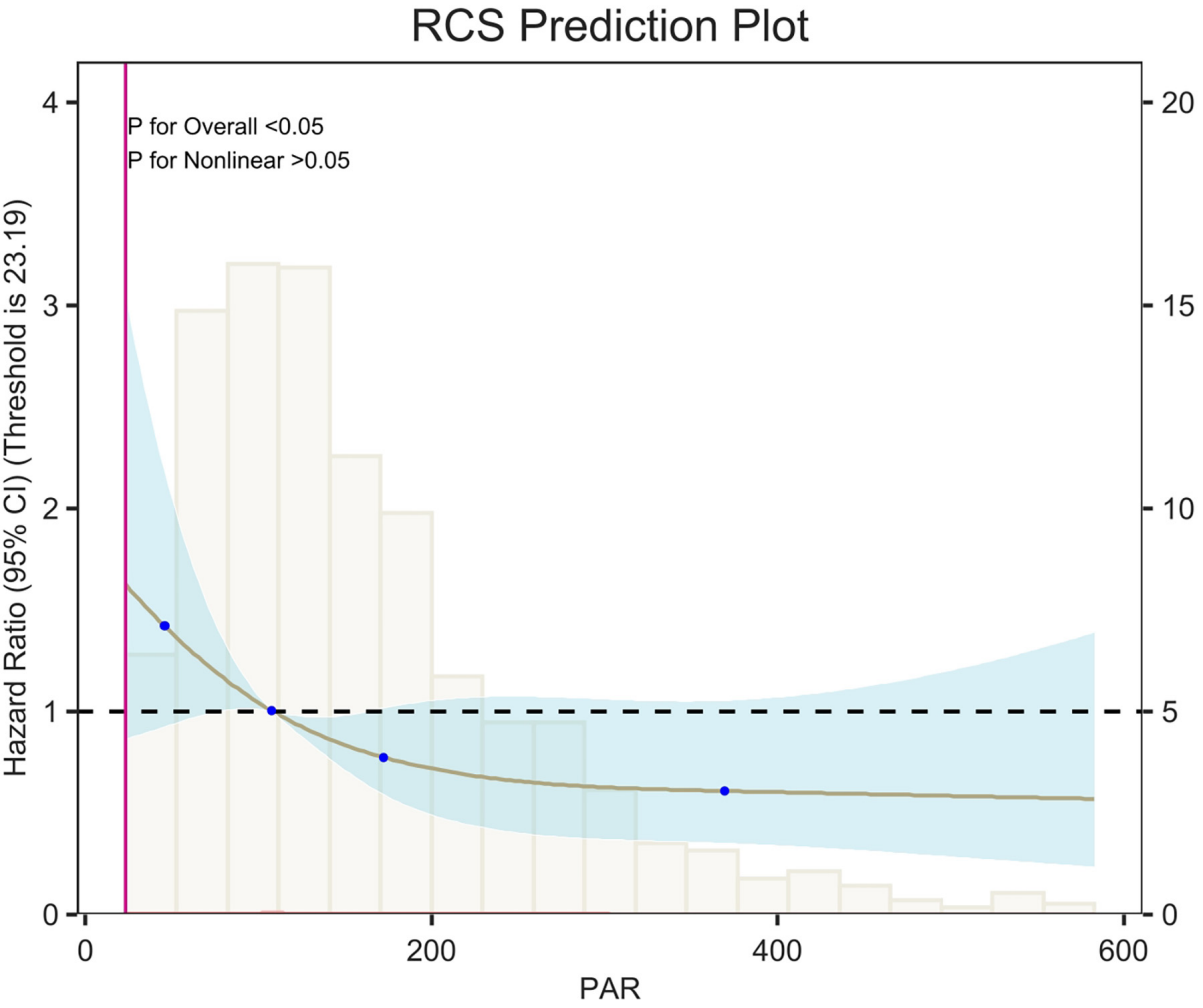
Restricted cubic spline (RCS) analysis of 14-day all-cause mortality. The curves show the adjusted estimated risk ratios, and the shaded bands show the 95% confidence intervals. The horizontal dashed line indicates a risk ratio of 1.0. HR, hazard ratio; CI, confidence interval.

**FIGURE 7:**
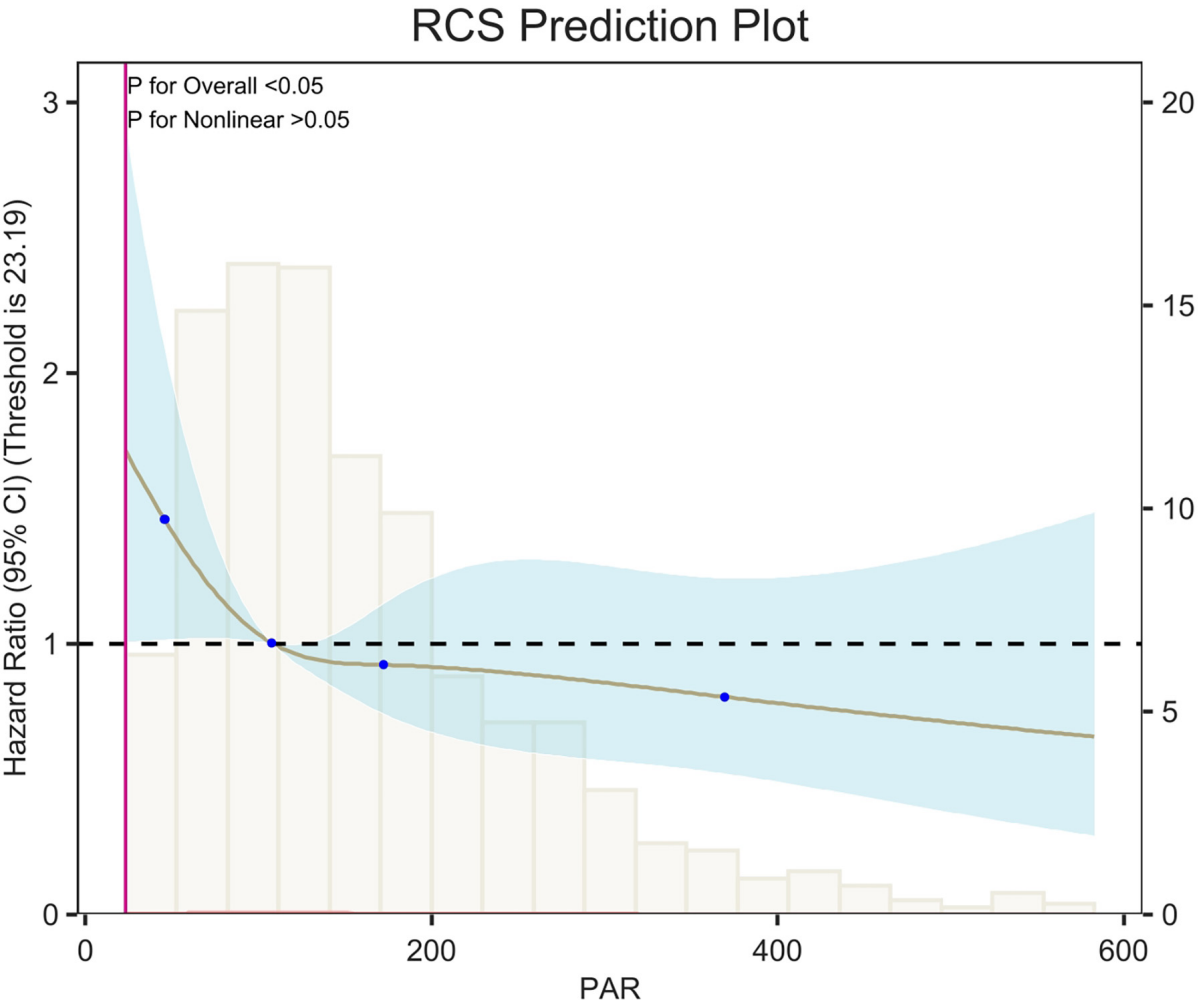
Restricted cubic spline (RCS) analysis of 28-day all-cause mortality. The curves show the adjusted estimated risk ratios, and the shaded bands show the 95% confidence intervals. The horizontal dashed line indicates a risk ratio of 1.0. HR, hazard ratio; CI, confidence interval.

Restricted cubic spline (RCS) analyses adjusted for age, sex, race, and weight showed an L-shaped linear correlation between population attributable risk (PAR) and risk of death (*p*-value < 0.05, *p*-Non-linear > 0.05) for 7-d, 14-d and 28-d all-cause mortality (Figures 5–7). The node counts in the RCS diagram are 47.008; 108.1875; 172.2140 and 368.8308. The threshold is 23.19. However, when PAR = 47.008, this represents the point at which the relationship between PAR and mortality risk begins to change. The risk of death decreased rapidly with increasing PAR. Then it leveled off gradually. Lower PAR is consistently associated with a poorer prognosis in patients with pulmonary embolism, and higher PAR may be a protective factor in the short term but may increase the risk of death in the long term.

## Subgroup analysis

**FIGURE 8:**
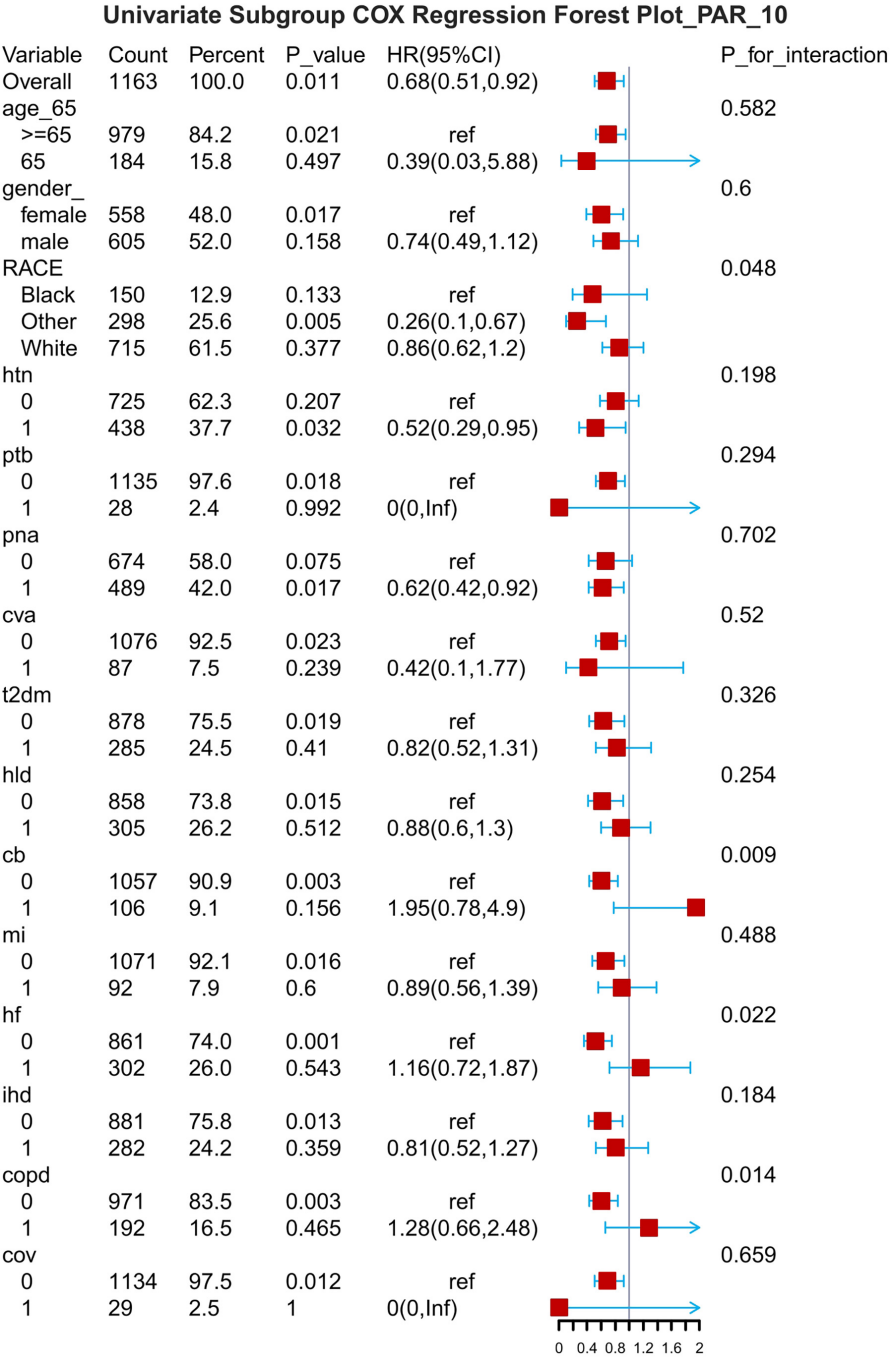
Subgroup forest plot for 7-day all-cause mortality. HTN, hypertension; PTB, pulmonary tuberculosis; PNA, pneumonia; T2DM, type 2 diabetes mellitus; T1DM, type 1 diabetes mellitus; HLD, hyperlipidemia; CB, chronic bronchitis; HF, heart failure; MI, myocardial infarction; IHD, coronary artery disease; COPD, chronic obstructive pulmonary disease; COV, coronavirus infection.

**FIGURE 9:**
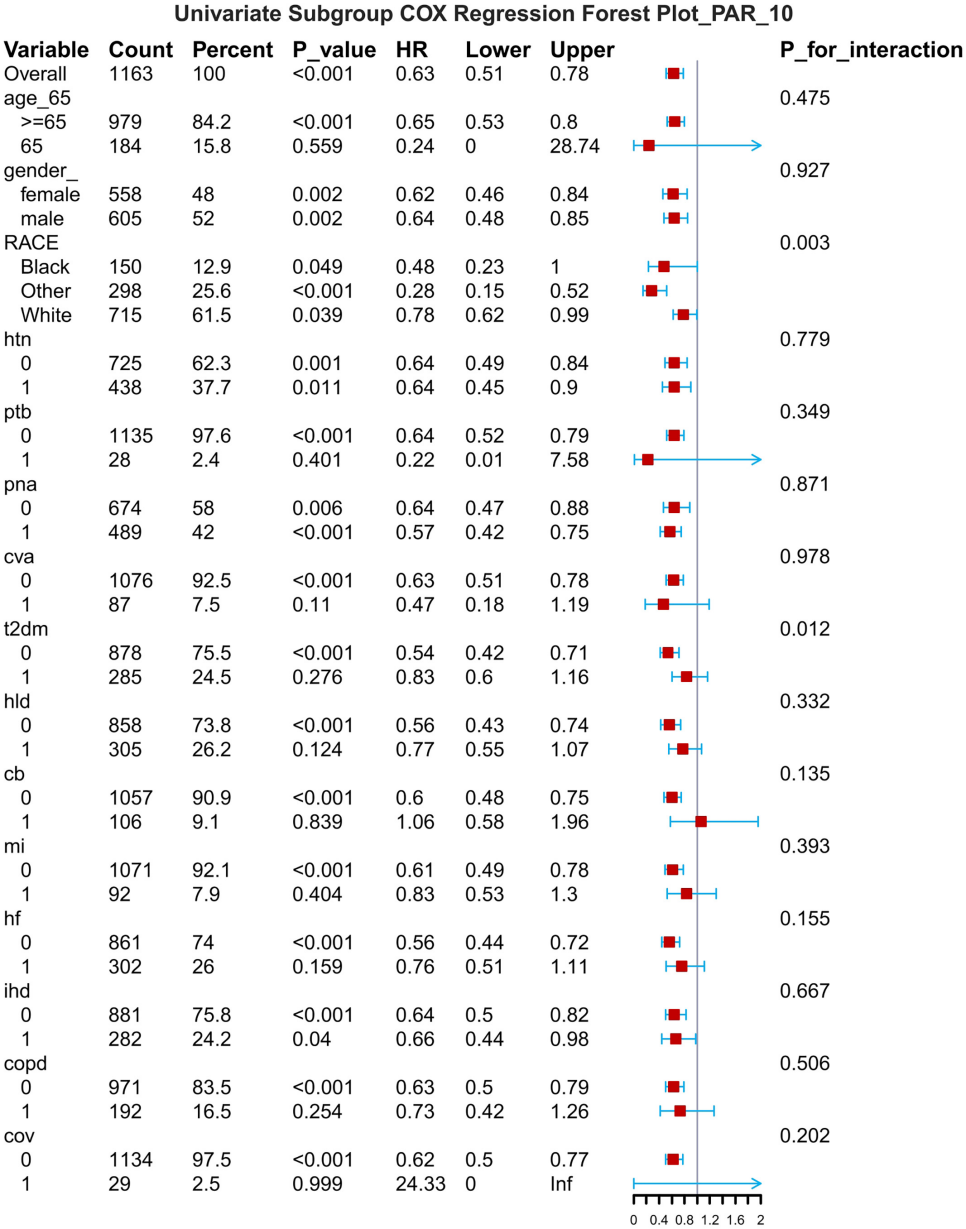
Subgroup forest plot for 14-day all-cause mortality. HTN, hypertension; PTB, pulmonary tuberculosis; PNA, pneumonia; T2DM, type 2 diabetes mellitus; T1DM, type 1 diabetes mellitus; HLD, hyperlipidemia; CB, chronic bronchitis; HF, heart failure; MI, myocardial infarction; IHD, coronary artery disease; COPD, chronic obstructive pulmonary disease; COV, coronavirus infection.

**FIGURE 10:**
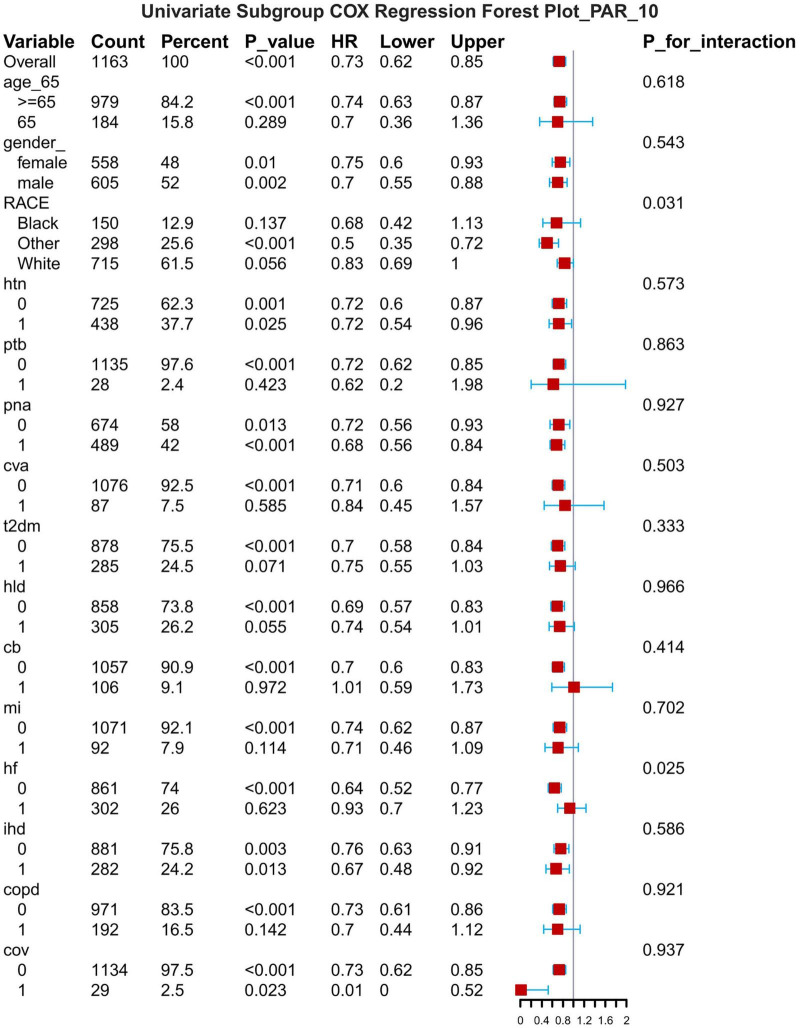
Subgroup forest plot for 28-day all-cause mortality. HTN, hypertension; PTB, pulmonary tuberculosis; PNA, pneumonia; T2DM, type 2 diabetes mellitus; T1DM, type 1 diabetes mellitus; HLD, hyperlipidemia; CB, chronic bronchitis; HF, heart failure; MI, myocardial infarction; IHD, coronary artery disease; COPD, chronic obstructive pulmonary disease; COV, coronavirus infection.

The results of the subgroup analyses are presented for 7-d, 14-d and 28-d all-cause mortality (Figures 8–10). In subgroups defined by age (under 65 years or over 65 years), sex (male or female), race, hypertension status and so on, PAR was significantly associated with the occurrence of outcomes.

## Boruta algorithm

**FIGURE 11:**
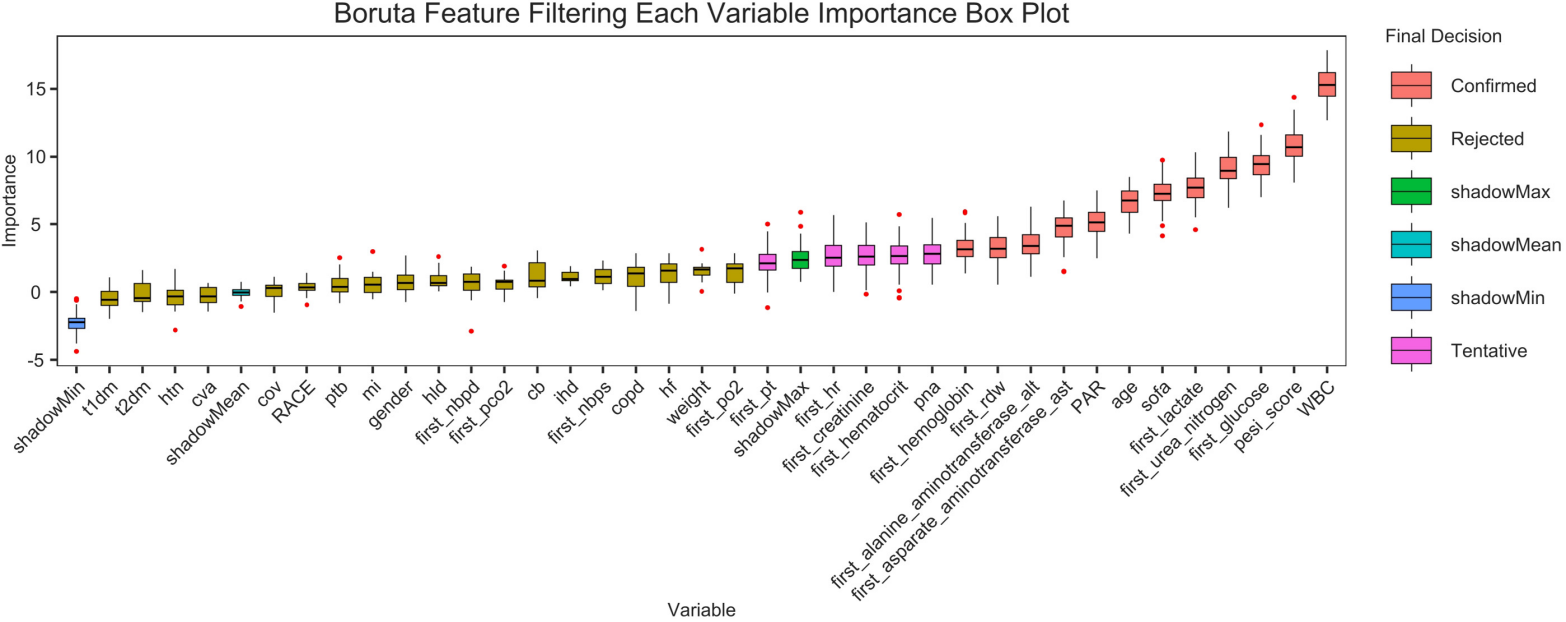
Feature selection based on the Boruta algorithm. The horizontal axis is the name of each variable, and the vertical axis is the *Z*-value of each variable. The box plot shows the *Z*-value of each variable during model calculation. The r boxes represent important variables, and the Yellow area represent unimportant variables.

Figure 11 shows the feature selection results based on the Boruta algorithm. Variables in the Red area are identified as important features, and variables in the Yellow area are unimportant features in the Boruta algorithm.

## Establishment and validation of the prediction model

**FIGURE 12:**
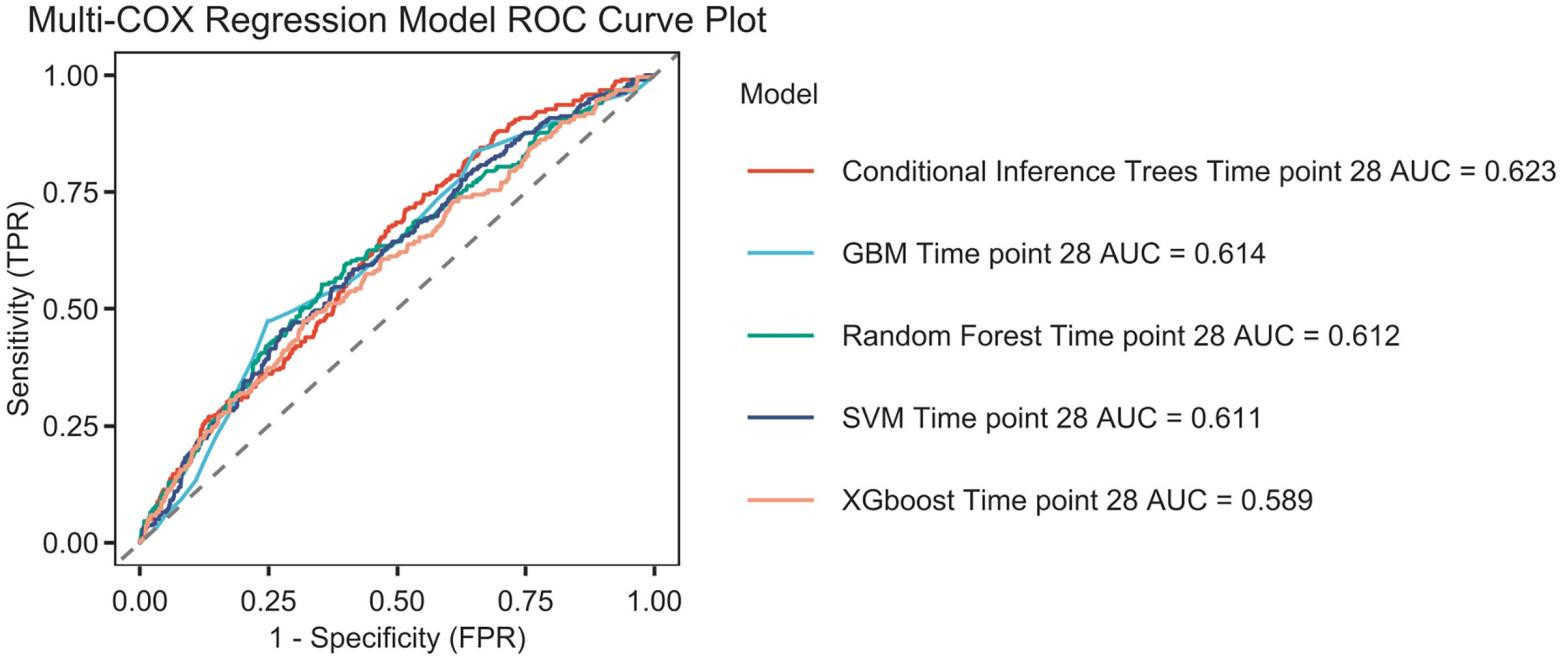
The Receiver Operating Characteristic (ROC) curves for machine learning algorithms are presented. The following algorithms are included: Conditional inference trees, Conditional inference trees survival learner, GBM (Gradient boosted trees) survival learner, Random forest time, Random forest survival learner, SVM (Support vector machines) survival learner, XGBoost (Extreme Gradient Boosted) survival learner, and time point 28. The area under the curve (AUC) is also reported.

Figure 12 shows the ROC curves of various models, and the model performance is indicated by the AUC values: Conditional inference trees: 0.623, GBM: 0.614, Random forest time: 0.612,SVM: 0.611,XGBoost (17):0.589.

## Discussion

This study preliminarily explored the predictive value of the platelet-to-albumin ratio (PAR) for short-term mortality in patients with pulmonary embolism using machine learning algorithms. As a composite indicator, PAR derives its clinical significance from the pathophysiological basis of its two components. An elevated platelet count may indicate acute inflammation and a tendency toward thrombosis. Conversely, hypalbuminemia reflects malnutrition, inflammatory states and hepatic dysfunction, and is closely associated with the severity of illness in critically ill patients. Although the discriminatory power of the single PAR indicator is limited (AUC = 0.623), it has a robust pathophysiological basis and is highly accessible and cost-effective in clinical practice. Therefore, PAR theoretically serves as a composite biomarker. It integrates thrombotic tendency, inflammatory status, and nutritional reserves. It potentially exhibits a certain correlation with short-term prognosis in patients with pulmonary embolism. This is the first study to investigate the relationship between PAR and adverse outcomes in patients with pulmonary embolism. As an exploratory study, its primary objective is not the immediate deployment of a clinical decision support system, but rather to validate the potential association between PAR (a novel, simple indicator) and PE prognosis through a data-driven approach. With an AUC value of 0.623, it is confirmed that this association is indeed present and not merely coincidental. This provides preliminary evidence for larger-scale, more in-depth studies to be conducted in the future. These findings were consistent across age, sex, and pneumonia and hypertension subgroups after adjusting for covariates in the study, demonstrating the robustness of the results. The Boruta algorithm (18) is a widely used feature selection method that determines which features are most important for predicting the target variable by modeling randomness. The results of the feature selection from the Boruta algorithm in this study indicate that PAR is located in the red zone. This suggests a correlation between PAR and the research objective. However, this does not imply that it is a decisive factor, as it may be affected by correlations between different data features. Secondly, a Cox regression analysis showed that lower PAR levels were linked to an increased risk of mortality within 28 days in patients with pulmonary embolism. This finding provides further support for the aforementioned perspective. We thus conclude that PAR serves as a predictor of 28-day all-cause mortality in patients with pulmonary embolism. The results confirmed that this association exists by incorporating acceptable variables into various machine learning algorithms. It can therefore be reasonably inferred that the platelet-to-albumin ratio is a potential predictor in this study.

### Relation to previous research

Previous studies have mainly focused on PAR in relation to IgA nephropathy (19), cancer-related diseases (20), and cardiovascular diseases (21). Regarding IgA nephropathy, studies have shown that patients with high PAR levels tend to exhibit more severe clinical manifestations and pathological lesions. Regarding cancer, PAR may serve as an independent predictor and prognostic factor. A previous study demonstrated that PAR and C2HEST score are independent risk factors for new-onset atrial fibrillation (NOAF) (14, 22) in patients with ST-segment elevation myocardial infarction.

### Pulmonary embolism pathogenesis

Essentially, when a deep vein thrombus dislodges (23), it embolizes the pulmonary arterial system. This triggers disproportionate ventilation and blood flow, a sudden increase in right ventricular afterload, and an imbalance in myocardial oxygen supply and demand (24). These factors can rapidly progress to obstructive shock or sudden cardiac death. The clinical manifestations of pulmonary embolism (1) (PE) are characterized by significant heterogeneity, ranging from asymptomatic episodic embolisms to high-risk massive embolisms (25). This heterogeneity results from the complex interplay of embolic load, underlying cardiopulmonary function, and neurohumoral compensatory capacity.

PAR and Pulmonary Embolism. However, elevated platelet (26) counts are not purely a compensatory response; rather, they reflect platelet overactivation, which is characterized by increased release of PF4 and sCD40L (4, 26). This overactivation exacerbates right ventricular afterload (27) by promoting the formation of neutrophil extracellular traps [NETs (28)] and pulmonary microvascular inflammation (29). Synchronized decreases in albumin levels suggest persistent activation of the coagulation-inflammation cascade. Albumin (5, 30) levels below 3.0 g/dL indicate vascular endothelial glycocalyx damage, resulting in capillary leakage and insufficient circulating blood volume. This leads to the amplification of hemodynamic collapse in right heart failure. As a negative acute-phase protein, low albumin levels directly reflect the systemic inflammatory state (elevated levels of IL-6/TNF-α) and are associated with the release of myocardial inhibitory factors (31).

## Impact of PAR and pulmonary embolism on clinical practice

As a simple and easily accessible indicator, PAR is expected to be used in clinical practice to assess the early risk of death in patients with pulmonary embolism (PE). Calculating PAR allows physicians to stratify patients more quickly. PAR essentially integrates the “hypercoagulability-inflammation-metabolic depletion” triad, which is more predictive than a single indicator. For patients with low PAR, which suggests a high risk of death, the treatment strategy can be adjusted over time. Examples include strengthening anticoagulation (32), considering thrombolytic therapy (33), and providing more active supportive therapy. However, the prognosis of a patient should not be determined by a single PAR indicator. In clinical practice, PAR should be used in conjunction with the patient’s clinical symptoms and signs, other laboratory tests, and imaging results to make a comprehensive assessment. More large-scale, prospective studies are needed to clarify PAR’s value in evaluating the prognosis of PE patients, optimize PAR’s application process, and improve its usefulness in clinical practice.

Our study shows that lower PAR is associated with an increased risk of 28-day and in-hospital all-cause mortality in critically ill patients with pulmonary embolism. This suggests that PAR can predict the risk of adverse events in a wider range of patients with pulmonary embolism and provide clinicians with a reliable indicator for diagnosing and treating critically ill patients with pulmonary embolism. For example, during rapid triage in the emergency department, a simple PAR-driven rule could quickly identify high-risk patients who require priority care. This could serve as a supplement to complex scoring systems at the front end.

## Limitations of the study

This study had a relatively small sample size, comprising patients with similar demographic characteristics and treatment strategies. The issues of missing data may have impacted model performance to some extent. Even when multiple interpolation strategies are employed, the absence of certain key variables can still introduce bias. This can limit the capacity of machine learning models to learn, particularly complex tree models, making them prone to overfitting or underfitting. Consequently, this affects their ability to generalize. Based on its current performance, the model constructed in this study may not yet be robust enough for direct application in clinical practice as an independent decision-making tool. The bottleneck in predictive performance is probably due to the information content of the predictors rather than the modelling algorithm itself. Nevertheless, it establishes a preliminary evidence base and source of hypotheses for the further validation of PAR’s clinical value.

## Final comments

Platelet-to-albumin ratio fills the gap in the “inflammation-metabolism dimension” of current pulmonary embolism (PE) risk stratification, offering the advantages of low cost and rapid accessibility. PAR’s value lies in its ability to provide an early warning of mortality risk and reveal the underlying driving mechanisms of right heart failure: persistent thrombotic inflammation and metabolic depletion. Ultimately, lower PAR is significantly associated with an increased risk of adverse events, irrespective of the presence of myocardial infarction. PAR can be used to predict adverse outcomes in patients with severe pulmonary embolism. However, researchers still need to conduct multicenter prospective studies to validate these results. PAR can be used to predict adverse outcomes in critically ill patients. Therefore, I believe that the next step should be to develop a multimodal predictive model that integrates PAR, clinical characteristics, imaging data and dynamic trends, in order to provide a more comprehensive risk assessment for critically ill patients. A more pragmatic pathway toward clinical application may be found by exploring the combined value of PAR alongside other emerging biomarkers, such as myocardial injury markers and inflammatory cytokines. This could be achieved by integrating them with established clinical scoring systems and machine learning model outputs.

## Data Availability

The raw data supporting the conclusions of this article will be made available by the authors, without undue reservation.
